# The Chemical Validation and Standardization Platform (CVSP): large-scale automated validation of chemical structure datasets

**DOI:** 10.1186/s13321-015-0072-8

**Published:** 2015-06-19

**Authors:** Karen Karapetyan, Colin Batchelor, David Sharpe, Valery Tkachenko, Antony J Williams

**Affiliations:** Royal Society of Chemistry, US Office, 904 Tamaras Circle, Wake Forest, NC 27587 USA; Thomas Graham House, Science Park, 290 Milton Road, Cambridge, UK; Environmental Protection Agency, Research Triangle Park, NC USA

**Keywords:** Chemistry, Validation, cvsp

## Abstract

**Background:**

There are presently hundreds of online databases hosting millions of chemical compounds and associated data. As a result of the number of cheminformatics software tools that can be used to produce the data, subtle differences between the various cheminformatics platforms, as well as the naivety of the software users, there are a myriad of issues that can exist with chemical structure representations online. In order to help facilitate validation and standardization of chemical structure datasets from various sources we have delivered a freely available internet-based platform to the community for the processing of chemical compound datasets.

**Results:**

The chemical validation and standardization platform (CVSP) both validates and standardizes chemical structure representations according to sets of systematic rules. The chemical validation algorithms detect issues with submitted molecular representations using pre-defined or user-defined dictionary-based molecular patterns that are chemically suspicious or potentially requiring manual review. Each identified issue is assigned one of three levels of severity - Information, Warning, and Error – in order to conveniently inform the user of the need to browse and review subsets of their data. The validation process includes validation of atoms and bonds (e.g., making aware of query atoms and bonds), valences, and stereo. The standard form of submission of collections of data, the SDF file, allows the user to map the data fields to predefined CVSP fields for the purpose of cross-validating associated SMILES and InChIs with the connection tables contained within the SDF file. This platform has been applied to the analysis of a large number of data sets prepared for deposition to our ChemSpider database and in preparation of data for the Open PHACTS project. In this work we review the results of the automated validation of the DrugBank dataset, a popular drug and drug target database utilized by the community, and ChEMBL 17 data set. CVSP web site is located at http://cvsp.chemspider.com/.

**Conclusion:**

A platform for the validation and standardization of chemical structure representations of various formats has been developed and made available to the community to assist and encourage the processing of chemical structure files to produce more homogeneous compound representations for exchange and interchange between online databases. While the CVSP platform is designed with flexibility inherent to the rules that can be used for processing the data we have produced a recommended rule set based on our own experiences with the large data sets such as DrugBank, ChEMBL, and data sets from ChemSpider.

## Background

The accurate representation and identification of chemical structures is one of the main tasks in the field of cheminformatics. There are multiple available representation formats for a chemical compound including a systematic name (e.g., IUPAC Name), a molfile connection table [1], and string representations such as various flavors of SMILES [2] and the InChI [3]. While we would expect this to be a mature technology, at this time only organic molecules are well covered in terms of exchange formats and standards such as InChIs with support for other structure forms such as Markush structures and organometallics being incomplete. Unfortunately, many scientists or programmers attempting to deal with a collection of chemical compounds in electronic format generally do not possess either sufficient chemistry or cheminformatics background and may often introduce errors in chemical representation. For example, different flavors of SMILES (generic, isomeric, canonical, etc.) from different vendors can be incorrectly interchanged and/or treated as absolute SMILES. Similarly, users may not understand the difference between standard and non-standard InChI strings and may treat them interchangeably. In some cases users may attempt to generate a chemical structure from an InChI believing that the result will be the equivalent of the original structure contained in the molfile but this is often not the case. Users of such algorithms do not necessarily fully appreciate the differences between these formats and the inter-exchange between them can introduce inconsistencies, breaking the correct relationships between synonyms, SMILES, InChI, and the structural data in the form of molfile.

Further complications can arise when a chemical record is displayed by different software packages as often the software has different default settings and what a user sees on the computer screen may be wrongly interpreted relative to what is contained in the actual structural data file.

**Fig. 1:**
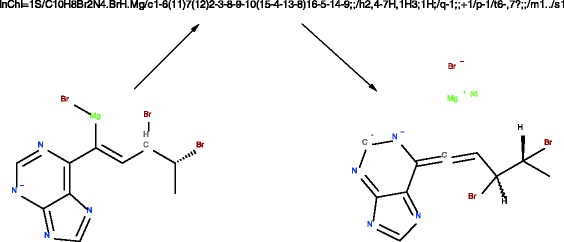
A depiction of how a chemical structure can change between InChI generation and InChI conversion. The original structure on the left was the hypothetical structure input to the InChI algorithm to generation the InChI string shown at the top of the figure. The conversion of the InChI string back to a visual form of the structure using Accelrys Draw resulted in changes including disconnection of the metal, changing the bonds and the ionization state of the halogen

To help to solve the problem of proliferation of multiple non-interchangeable identifiers InChI was developed under the guidance of an IUPAC sanctioned committee as an open structure identifier. The generation of InChIs involves the normalization of the original structure, and its canonicalization and serialization [4]. Standard InChI normalization involves disconnecting metals, the removal/addition of protons, simple tautomer detection/canonicalization and the conversion of relative stereo to absolute, etc. Therefore, an InChI does not actually represent the original structure but its normalized version and an InChI string is not really intended for backward structure generation as it can lead to a molecule different from the one that was used for InChI generation (see Fig. 1). Often this is overlooked and thus there is a potential loss of information when using an InChI as the primary source of the structure rather than the original connection table in a molfile. An example of a hypothetical molecule that was converted to an InChI and then back to a structure using the Accelrys Draw [5] structure drawing application is presented on Fig. 1.

Some chemical structure drawing programs allow users to specify double bond stereochemistry in an “either” form (commonly represented as a crossed bond) or as specifically defined E/Z stereo. However, users rarely use the “either” representation of the unknown double bond for accurate representation. Without an “either” bond InChI algorithm appropriately deduces the stereochemistry of the double bond based on the Cartesian coordinates. This often brings an additional inconsistency between what was intended to be drawn and what is encoded into the InChI.

Another common caution is in regards to structures with partially defined stereo versus unknown stereo. The standard InChI does not distinguish between undefined and explicitly marked “unknown” sp^3^ stereo. Therefore, an attempt to convert backwards from a standard InChI would guarantee the loss of stereo information.

Relative stereochemistry that is possible to define in a molfile by omitting the chirality flag would be treated by standard InChI normalization as absolute and converting back from that InChI to a molfile would produce absolute stereo. The handling of advanced stereochemistry will hopefully be treated in a future version of the InChI standard.

When dealing with data sets that contain combinations of connection tables, InChIs, SMILES, and chemical names one of the important questions to ask is which of these forms of the chemical structure is expected to be the primary source of structural data. Often it is the connection table and all other representations or identifiers (names, SMILES, InChI, etc.) were supposedly derived from it. In such cases it would make sense to cross-validate all these other representations with the connection table. However, data owners rarely do such validations and thus data is being propagated to commercial or public databases as is, especially with regards to the miss-association of chemical compounds with their associated chemical names [6, 7]. Sometimes InChI, SMILES or chemical names (both systematic and common names) are the primary reference of the structure and in such cases other structural identifiers could be cross validated against them.

Based on our experiences as hosts of the ChemSpider database [8–10], data owners commonly do not pre-validate or emphasize the quality of their data. This has historically led to fairly high rejection rates of data at the time of deposition onto ChemSpider and, when filtering systems were not in place during the early phases of the project, to the introduction of poor quality data onto the platform. Such unwillingness to share the responsibility for data quality has ultimately affected many of the online chemistry databases especially since many source from each other’s content. We contend that even simple validation exercises that would at least cross-match any given structure connection table with the depositor’s synonyms, SMILES or InChIs would have had an overall positive influence on data quality. As a result of the growing proliferation of online chemistry databases we believe that there is a growing need to develop an open and free platform that could intake chemical records and generate validation messages in a categorized and concise manner such that data submitters could review the data according to this validation process. While it would be ideal to have a single community agreed upon set of rules that could be implemented to satisfy the needs of all data providers we are cognizant of the fact that this is a significant challenge. However, this is no different really than the set of assumptions that underpin the Standard InChI that is presently utilized in the majority of databases, with one specific caveat. The validation and standardization of chemical compound datasets allow the user to review the impact of the process via visualization of the impact on the structure representation, a process far more amenable to a scientist than the interrogation of an alphanumeric text string. Also, the process of validating and standardizing the data *prior* to the generation of InChIs, whether standard or non-standard, would be a more ideal process. Nevertheless, acknowledging that different database hosts may have different needs in regards to the processing of their data sets an open system should be designed in a manner that allows for either the expansion of, or exclusion of specific rules.

A specific driver for the need for an open platform for the validation and standardization of chemical compound datasets is the participation of our cheminformatics team as part of the Open PHACTS project. Open PHACTS [11, 12] is a semantic web project with the primary charge of meshing together public domain chemistry and biology datasets and driven primarily by a commitment to open data and open standards. As part of the project our team was specifically tasked with producing a system that could manage the chemical compounds collection that would be a part of the Open PHACTS open data collection. The criteria for selection of the datasets to populate the Open PHACTS chemical registration service (CRS) were focused primarily on utility of the underlying data, popularity and regard for the data sets within the community and as a result of a polling of members of the consortium. Datasets of interest include ChEMBL [13, 14], ChEBI [15] and DrugBank [16, 17]. Our previous experiences of handling these various datasets when assembling them as contributing data sources into ChemSpider indicated some data quality issues across the various data sources but, more importantly, the need for pre-processing each of the relevant data sources into a standardized form prior to populating the CRS. The details of the CRS will be reported in a separate publication and the focus of this article will be in regards to our approach to processing the various data sets using a Chemical Validation and Standardization Platform.

## Results

**Fig. 2:**
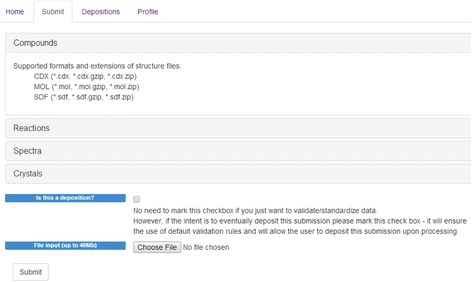
The upload screen for CVSP – the various supported file formats are listed. Compressed file formats (ZIP and gzip) are supported

The idea of having a free platform that would validate chemical records for scientific article authors, data depositors and curators of ChemSpider, and data set owners has been the primary driver for the cheminformatics team at the Royal Society of Chemistry to develop such a platform. The Chemical Validation and Standardization platform (CVSP) has been developed using the GGA’s Indigo and OpenEye cheminformatics toolkits [18, 19] and a number of in-house libraries. CVSP validation works record by record and takes input files in MOL, SDF, and ChemDraw CDX formats. For ease of use compressed sets of files in gzip and zip formats are also supported. The input screen is shown in Fig. 2. Each record is expected to have a connection table that is considered as the primary structure representation and all other available annotations within the record (InChI and SMILES) are considered as secondary sources. Both the primary structure source (the connection table itself) and its relationship to the secondary structure sources are validated.

**Fig. 3:**
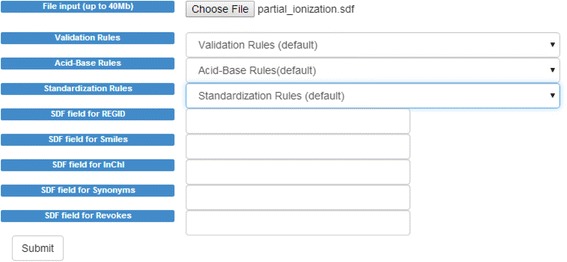
The SDF upload screen for CVSP

For SDF files users may map the SDF fields for additional validation. For example, in SDF they can map their external identifiers to CVSP’s REGID field, their SMILES to CVSP’s “SMILES” field, etc. Having these fields mapped allows CVSP to cross-validate them with the structure in the molfile (Fig. 3).

Before submitting the file users have to select the appropriate rule sets. To run complete CVSP validation users have to select the default rule sets for partially ionized acid/base pairs and validation modules. If standardization is required then the standardization rule set has to be set to default as well. Note that rules with the “default” label are the default platform-wide CVSP rules.

The chemical validation algorithms detect issues with the submitted molecular representations using pre-defined dictionary-based molecular patterns that are chemically suspicious or potentially could require manual review. Each identified issue is assigned one of three arbitrary levels of severity - Information, Warning, and Error – in order to conveniently inform the user of the need to browse and review subsets of their data. The validation process includes the validation of atoms, bonds, valences, and stereo. Standard InChIs are generated and assigned to each individual record according to the submitted connection table. As discussed earlier the standard form of submission of collections of data, the SDF file, allows the user to map the data fields to predefined CVSP fields for the purpose of cross-validating associated SMILES and InChIs with the connection tables contained within the SDF file.

**Fig. 4:**
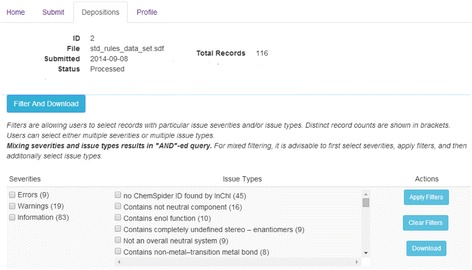
The filtering and download user interface screen for processing of the results set from CVSP

The resulting processed file can then be reviewed and records filtered according to various severities and issue types and subsets of the data can be downloaded. This may make it easier for a scientist to handle the data in software tools of their choosing and prepare the data for filtering and revalidation in CVSP. The filtering selection and export screen is shown in Fig. 4.

### Creating user validation rules based on SMARTS

**Fig. 5:**
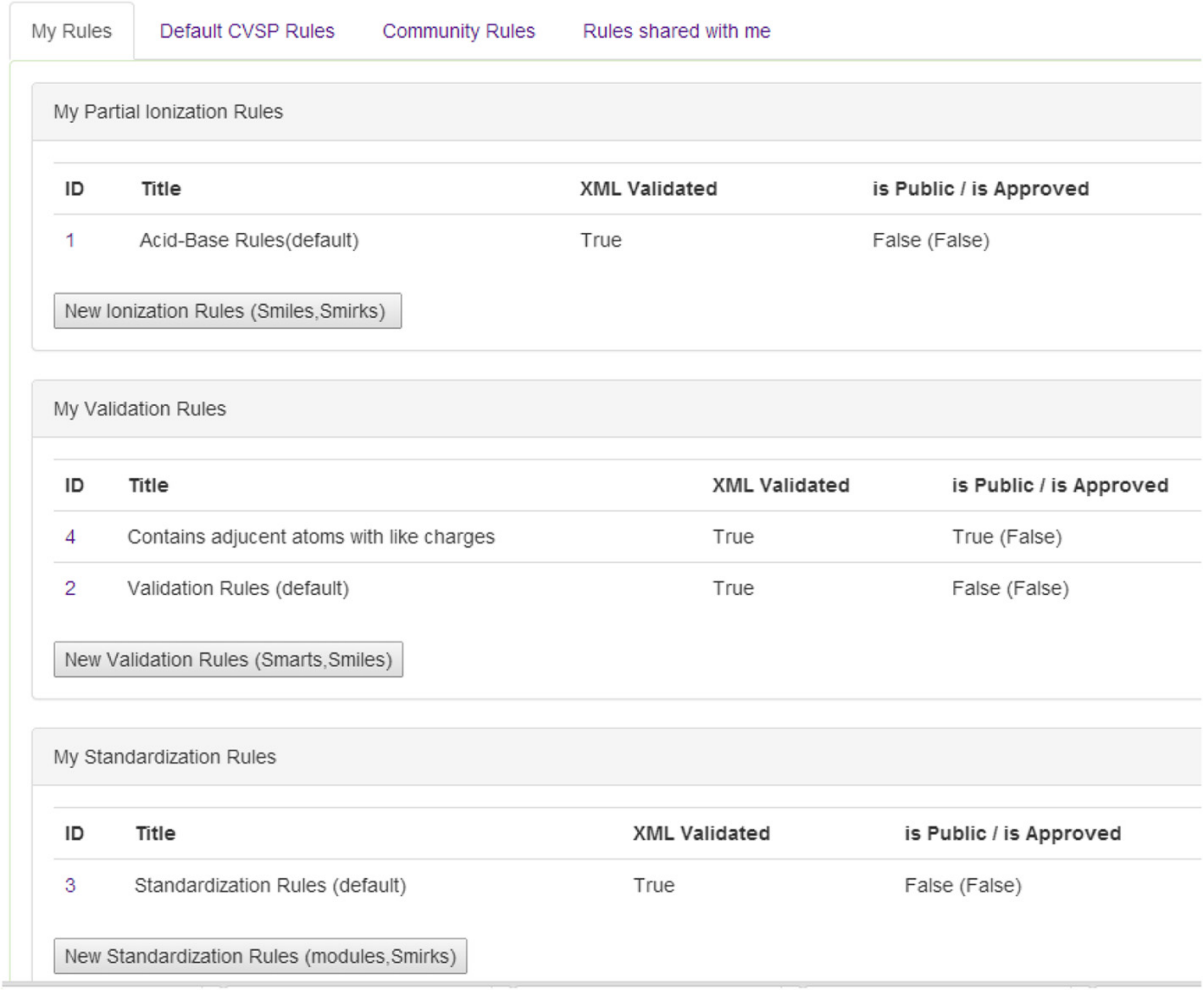
XML rules under user Profiles

Under the profile tab (Fig. 5) user will see 4 tabs:

- “My rules”
  CVSP allows users to define their own validation rules via an XML file. To create an XML file a user can either clone the default CVSP XML rule set and then modify it or create rules set from scratch. By creating their own rules users can use them when submitting files and they can also share rules by making their rules public (which requires approval of a CVSP curator).
- “Default CVSP Rules”
  This is the default unmodifiable platform-wide set of rules. Users can review and clone these rules. Cloned rules can be modified and are private to users.
- “Community Rules”
  These are rules that were shared by other CVSP users and were approved by a CVSP curator. Even though curators do their best to validate shared user content the team is not responsible for the deposited community rule set. Users are welcome to submit feedback regarding any issues via a “Feedback” button on the top right hand side of the CVSP web page.
- “Rules shared with me”
  These are rules that were specifically shared with other user(s) (thus private between certain users). This particular configuration is not fully implemented as yet the final implementation is dependent on community feedback.

Validation XML rules have to have a specific format as defined in the example below.

The rules themselves can be simple (one SMARTS per rule) or complex (AND-ed or OR-ed SMARTS). Each rule should have a severity tag “Warning”, “Information”, or “Error” (depending on what severity CVSP should assign to this rule). An attribute “message” should be a concise topic describing the rule whereas the attribute “description” should be a bit more informative. Inside of the severity tag should be a “test” tag with “name” and “param” attributes. The attribute “name” should always be “SMARTStest” as only SMARTS tests are supported at present. The attribute “param” should include the actual SMARTS string. The format of the user XML content is validated but it is up to the user to define the SMARTS appropriately.

Acid–base competitive ionization rules are defined in a different XML format.

Each “acidgroup” tag has to have the attributes “rank”, “acid”, “base”, “acid2base”, “base2acid”. Users that would like to develop their own competitive ionization rules would need to rank rules and for each acid and base define the SMARTS and also for each acid–base transformation define the SMIRKS.

### Warnings, errors and informational messages

The connection table validations include, but are not limited to the checks listed below.

**Fig. 6:**
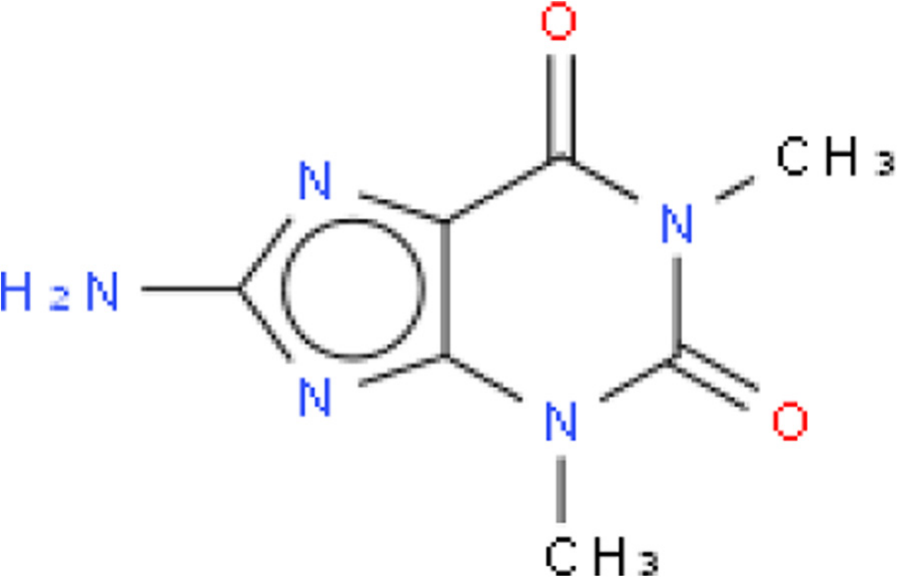
Not unique dearomatization

**Fig. 7:**
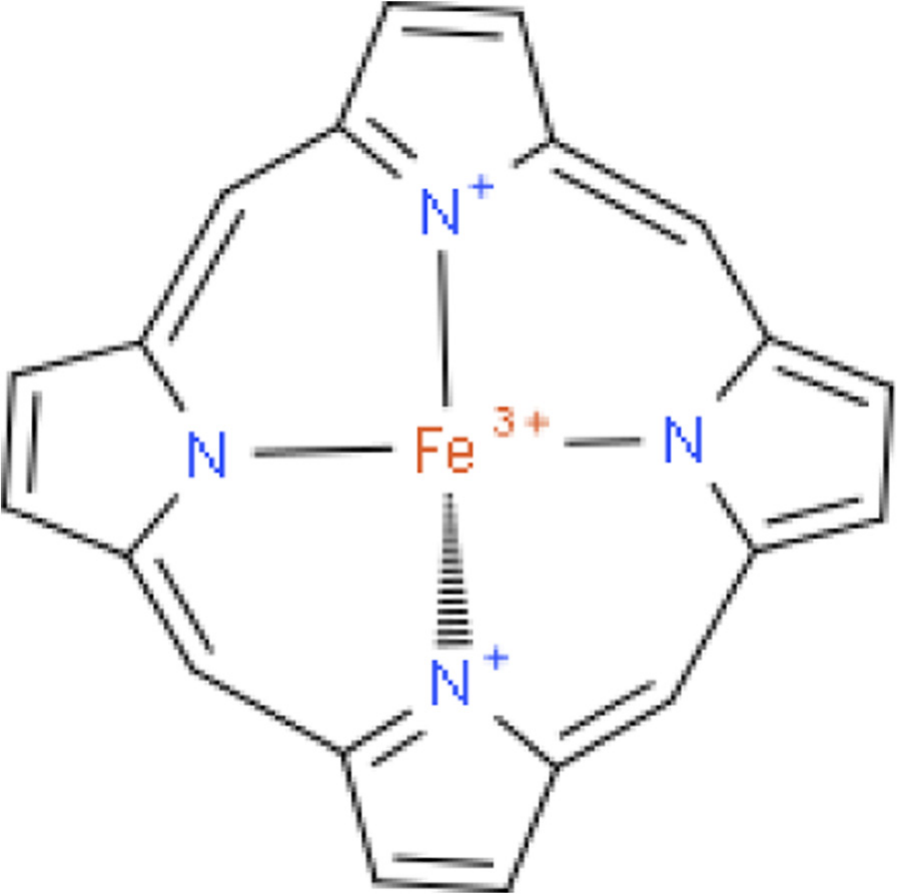
Direction of stereo bonds makes no sense

**Fig. 8:**
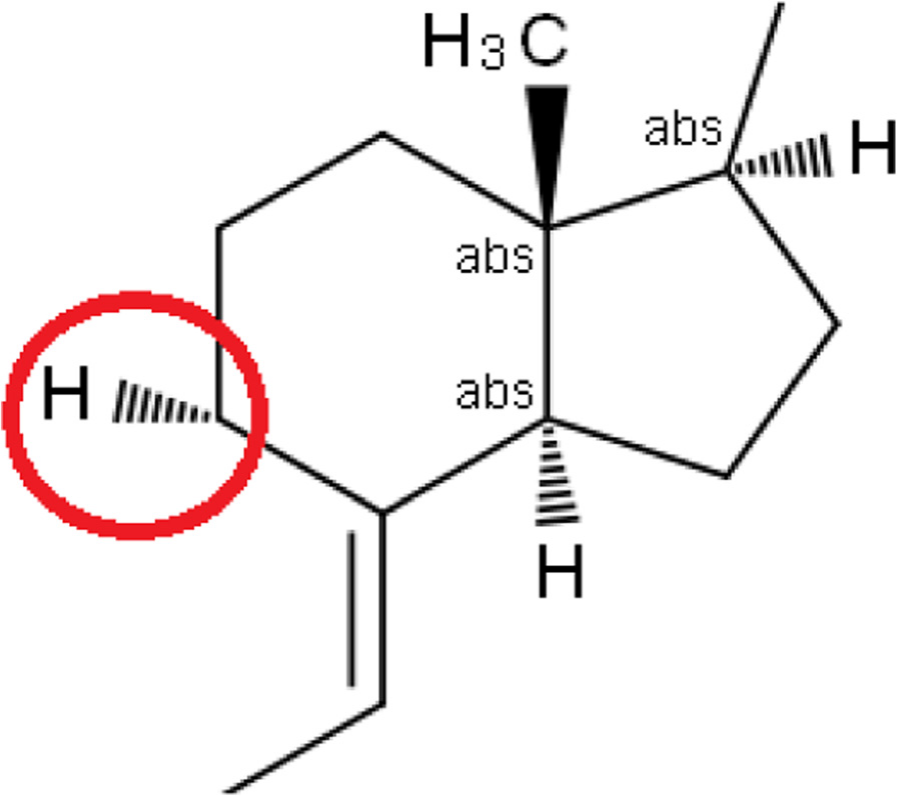
Non stereo center is marked with stereo bond

**Fig. 9:**
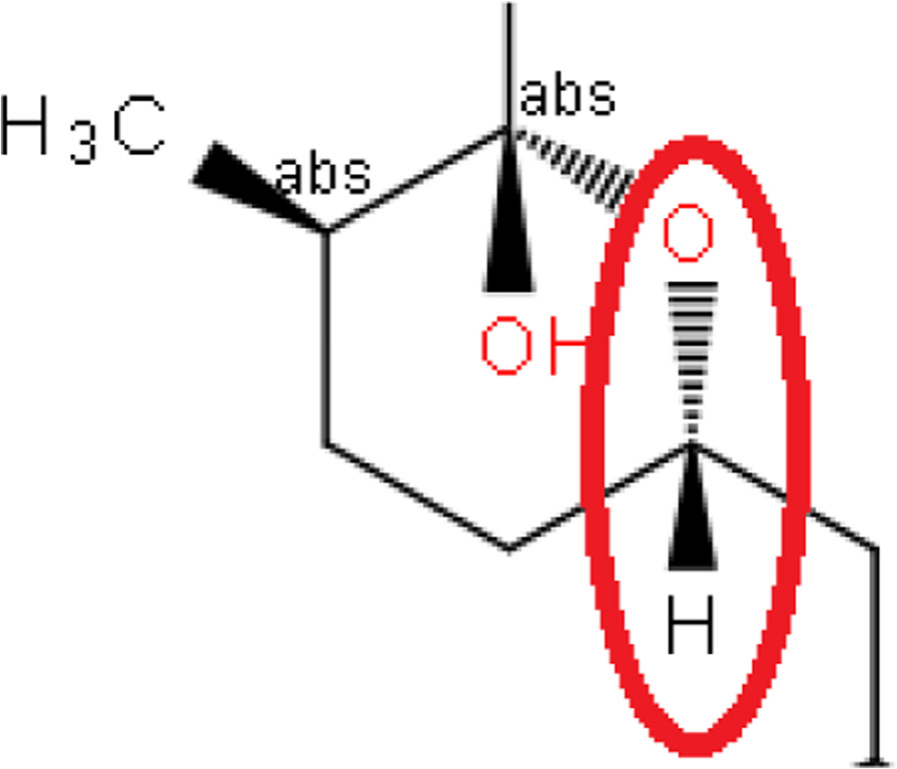
Stereo center has both up and down bonds

**Fig. 10:**
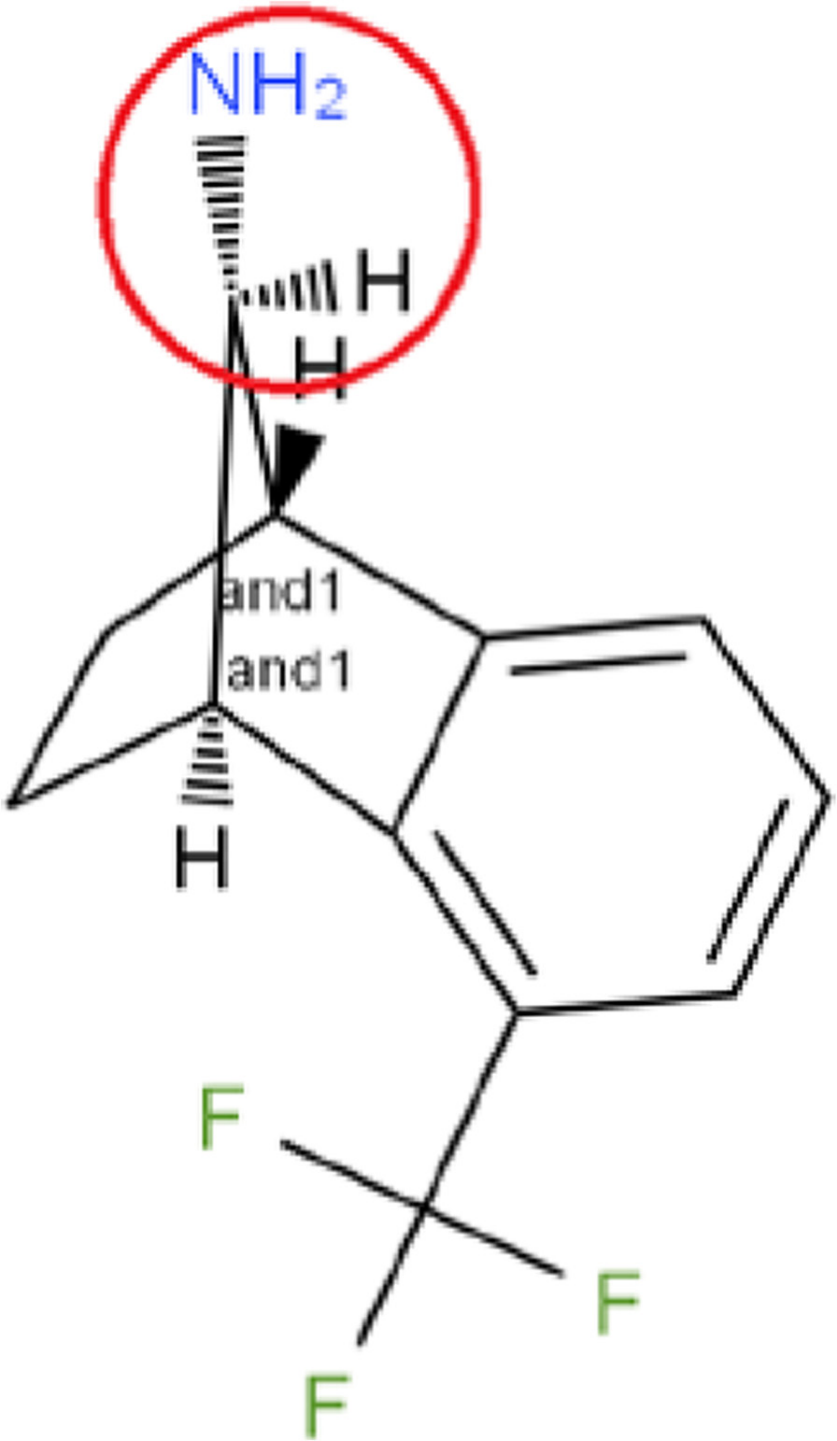
Two wedged or to hashed bonds at same center

**Fig. 11:**
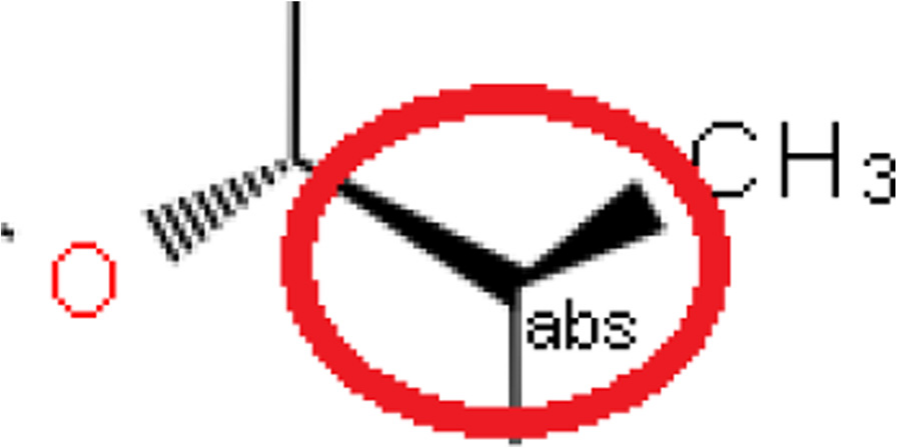
Same atom is destination and the origin of stereo bonds

Some of the “Error” types:

- invalid atom symbols (query atom)
- suspicious/unusual valences
- query bonds
- dearomatization is not unique, cannot restore hydrogens, e.g., Fig. 6
- direction of stereo bond makes no sense, e.g., Fig. 7
- angle between stereo bonds is too small
- a non-stereo center is marked with stereo bonds, e.g., Fig. 8
- both up and down stereo bonds are located at the same stereo center, e.g., Fig. 9
- two up or two down bonds on the same atom, e.g., Fig. 10
- up or down bond points from the atom, e.g., Fig. 11

Some of the “Warning” types:

- fragments such as methane, ammonia, water, elemental sulphur or boron are detected
- more than one radical center is detected
- structure contains unusual valence
- duplicate molecules
- “SMILES does not match the structure”: This warning is raised when the InChI generated from an input SMILES does not match the InChI generated from the connection table
- “InChI does not match the structure”:

Depositor-provided InChI does not match the InChI generated from the connection table

**Fig. 12:**
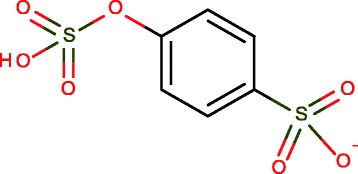
Partially ionized molecule

**Fig. 13:**
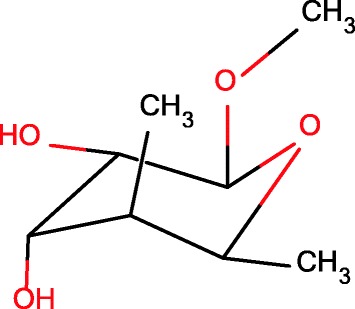
Howarth projection of monosaccharide

Some of the “Info” types:

- Covalent bonds connecting metals to non-metals
- Overall charge is non-zero
- Existence of certain functional groups (e.g., enol, non 1H-tetrazole, N = C-OH, N#N = N)
- Adjacent atoms with like charges
- Completely or partially undefined sp3 stereocenters. Currently 4 categories are detected and flagged:
  - ○ “partially undefined stereo – epimers” for molecules with at least one defined stereocenter and a single undefined/unknown stereocenter,
  - ○ “partially undefined stereo – mixtures” for molecules with at least one defined stereocenter and more than one undefined/unknown stereocenters,
  - ○ “completely undefined stereo – enantiomers” for molecules with no defined stereocenter and single undefined/unknown stereocenter
  - ○ “completely undefined stereo – mixtures” for none of defined stereocenters and at least one undefined/unknown stereocenters
- Double bond explicitly marked by user as unknown (cross bonds)
- Strongest acid not ionized first in partially-ionized system (see ranking of acids in Table 2), e.g., Fig. 12
- Contains a perspective Haworth formula, e.g., Fig. 13
- Contains 3D coordinates

## Methods

The platform uses the Indigo cheminformatics library [18] to handle low-level cheminformatics tasks such as molecule manipulation or basic stereochemistry interpretation. We have written our own C# code to process ChemDraw files, replacing the OpenBabel methods [20] described by Day *et al.* [21]. The initial set of validation rules consisted of those presented in Day *et al.* 2012 and the structure depiction rules found in the Substance Registry System document issued by the Food and Drug Administration [22]. We have extended these based on our experience of dealing with chemical structures in ChemSpider, journal article author-supplied ChemDraw files, and the stereochemical errors issued by Indigo.

In order to detect substructures in general and metal–non-metal bonds in particular we used pre-compiled SMARTS strings. To make it easier to implement the rules we established a set of abbreviations that enable many atoms to be specified at once. We list a sample of these in Table 1.

**Table 1:** Example SMARTS abbreviations and how they are interpreted by the code

Abbreviation	Interpretation
{NM}	Non-metals less carbon (here He, B, N, O, F, Ne, Si, P, S, Cl, Ar, Ge, As, Se, Br, Kr, Sb, Te, I, Xe, Po, At)
{M}	Metals (everything else)
{Pn}	Pnictogens (here P, As, Sb)
{Hal}	Halogens (here F, Cl, Br, I)
{M_V6}	Metals with maximum valency 6 (Cr, Mo, W, Mn, Pt)
{TM}	Transition metals
{TM^Hg}	Transition metals apart from mercury (needed for FDA rules)
{M_ + 1}	Metals with a charge of +1.

Another part of the FDA recommendations that can be readily handled with SMARTS detection is competitive ionization. The FDA recommends that in the case of ionized structures, for example salts, the most acidic protons be ionized first. In order to determine whether this is the case this we use the SMARTS strings and rankings in Table 2.

**Table 2:** Rankings (smallest numbers indicating most acidic) and SMARTS strings to identify acid and base substructures for competitive ionization of molecules based on FDA 2007

Group	Acid SMARTS	Conjugated Base SMARTS	Rank
OSO3H	OS(=O)(=O)[O;H]	OS(=O)(=O)[O-]	10
SO3H	[!O]S(=O)(=O)[O;H]	[!O]S(=O)(=O)[O-]	20
OSO2H	O[S;D3](=O)[O;H]	O[S;D3](=O)[O-]	30
SO2H	[!O][S;D3](=O)[O;H]	[!O][S;D3](=O)[O-]	40
OPO3H2	OP(=O)([O;H])[O;H]	OP(=O)([O;H])[O-]	50
PO3H2	[!O]P(=O)([O;H])[O;H]	[!O]P(=O)([O;H])[O-]	60
CO2H	C(=O)[O;H]	C(=O)[O-]	70
Arom-SH	c[S;H]	c[S-]	80
OPO3H-	OP(=O)([O;H])[O-]	OP(=O)([O-])[O-]	90
PO3H	[!O]P(=O)([O;H])[O-]	[!O]P(=O)([O-])[O-]	100
Phthalimide	O = C2c1ccccc1C(=O)[N;H]2	O = C2c1ccccc1C(=O)[N-]2	110
CO3H	C(=O)O[O;H]	C(=O)O[O-]	120
α-carbon to NO2 group	O = N(O)[C;H]	O = N(O)[C-]	130
SO2NH2	S(=O)(=O)[NH2]	S(=O)(=O)[NH-]	140
OB(OH)2	OB([OH])[OH]	OB([OH])[O-]	150
B(OH)2	[!O]B([OH])[OH]	[!O]B([OH])[O-]	160
Arom-OH	c[OH]	c[O-]	170
SH aliphatic	C[SH]	C[S-]	180
OBO2H	OB([OH])[O-]	OB([O-])[O-]	190
BO2H	[!O]B([OH])[O-]	[!O]B([O-])[O-]	200
Cyclopentadiene	[CH2]1C = CC = C1	[C-]1C = CC = C1	210
Amide	C(=O)[NH2	C(=O)[N;H;-]	220
Imidazole	c1cnc[n]1	c1cnc[n-]1	230
Aliphatic OH	[CX4][OH]	[CX4][O-]	240
H at α-carbon to carboxyl	O = C[CH]	O = C[C-]	250
H at α-carbon to acetyl	OC(=O)[CH]	OC(=O)[C-]	260
H at sp carbon	C#[CH]	C#[C-]	270
H at α -carbon of sulfone group	CS(=O)(=O)C[CH]	CS(=O)(=O)C[C-]	280
H at α-carbon of sulfoxide	C[S;D3](=O)C[CH]	C[S;D3](=O)C[C-]	290
Amine	[CX4][NH2]	[CX4][N;H;-]	300
Benzyl	c[C;D4;H]	c[C;D3;-]	310
H at sp2 carbon	[CX3;H]	[CX3;-]	320
H at sp3 carbon	[CX4;H]	[CX3-]	330

**Fig. 14:**
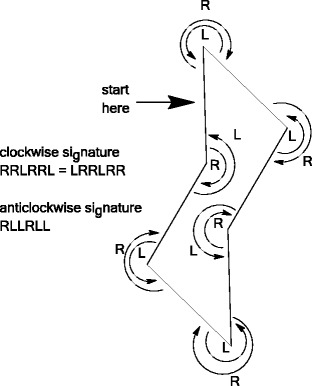
Depiction of the ring-walking algorithm. If we start at the topmost node on the ring and proceed clockwise recording whether there is a left turn (L) or a right turn (R) at every node, then we obtain a six-character “signature”. This indicates whether the hexagon is homotropous (all turns are the same), a chair, a boat or a yet more exotic shape

One particular challenge for molecular structure identifier generation is the depiction of carbohydrate stereochemistry, as, for example, the InChI algorithm [23] cannot cope with “perspective” drawings of pyranose rings in the chair conformation. A full description of our approach including standardization is out of scope for this paper; however the algorithm proceeds as follows. The Indigo toolkit is used to detect the unfused six-membered rings in the molecular structure, the shapes of which are then classified using the ring-walking algorithm depicted in Fig. 14, which identifies a “signature” for each ring based on whether on walking around the ring you take a left or a right turn at each node. Table 3 shows the eight possible shapes for hexagons along with common names, where they exist, and a small selection of signatures. The code compares the hexagon signature against all cyclic permutations of both a left-first and right-first “canonical” signature. By cyclic permutations we mean that, for example, LLLRRR = LLRRRL = LRRRLL = RRRLLL = RRLLLR = RLLLRR = LLLRRR. We also use this code to detect L-pyranose rings as in many cases these are the result of the author having inadvertently mirrored the ring (John Blunt, personal communication).

**Table 3:** Selected hexagon signatures, names (if any) and graphical depictions

Signatures	Name	Example	Signatures	Name (if any)	Example
LRLLRR, RLRRLL	Boat		LLLRRR	Twist–boat	
LRRRRR,RLLLLL	Half chair		LRLRLR	—	
LLLLLL, RRRRRR	Homotropous		LRLRRR, RLRLLL	—	
LRRLRR, RLLRLL	Chair		LRLLRR, RLRRLL	—	

### Application of CVSP to specific data sets

In this work we review the results of the automated validation of DrugBank 3.0 [16, 17, 23] and ChEMBL version 17 [14]. Some of the results are listed in Table 4.

**Table 4:** Some comparison of DrugBank and ChEMBL datasets

	DrugBank	ChEMBL	Examples
Errors			
Query bonds	2	0	DB00115
Stereocenters: stereotypes of non-opposite bonds match	1	292	DB08128, CHEMBL1183153, CHEMBL1971333
Stereocenters: stereotypes of opposite bonds mismatch	2	2542	DB00877, CHEMBL1237110
Stereocenters: one bond up, one down	1	182	DB01590, CHEMBL552998, CHEMBL1237113
Stereocenters: implicit hydrogen near stereocenter	1	1	DB00910, CHEMBL2314995
Non-unique dearomatization	57	0	DB01705
Unknown atom symbol (“A”, “*” - polymers)	3	0	DB01344
Bad Valence (Indigo)	1	0	DB01747
InChI generation failed	4	2	DB03846, CHEMBL1770360
Warnings			
InChI does not match structure	36	N/A	DB00162
Name does not match structure	24	N/A	DB08346
SMILES does not match structure	48	N/A	DB00520
Contains only multiple instances of same molecule	0	25	CHEMBL607305
Not a neutral system	314	14337	DB00118, CHEMBL13045
Angle between bonds too small	2	164	DB00362, CHEMBL59973
Free carbon monoxide	0	5	CHEMBL108869
Unusual valence	49	119	DB01703, DB03492, CHEMBL2028143, CHEMBL2028140
Relative stereo (wedge or hash bonds but no chiral flag in molfile)	1183	151203	DB00140, CHEMBL1801886
More than one radical atom	2	4	DB04119, CHEMBL606910
Information			
Contains enol function	64	11898	DB00554, CHEMBL62289
Stereobond in ring	4	943	DB00877, CHEMBL1864961, CHEMBL1864961
Contain unknown stereobond	32	23451	DB00162, CHEMBL1866933
Contain metal-nitrogen bond	25	60	DB02003, CHEMBL611725
Contain partially undefined stereo	24	26862	DB00462, CHEMBL63248
Strongest acid not ionized first	3	164	DB04798, CHEMBL8056
Contains L-pyranose	185	5887	DB00199, CHEMBL66563
Contains metal-oxygen bond	32		DB00526, CHEMBL611725

When looking up molfiles behind registry identifiers reported in Table 4 please make sure you use the appropriate versions of data for each data source, e.g., for ChEMBL you would need version 17 downloadable at:

ftp://ftp.ebi.ac.uk/pub/databases/chembl/ChEMBLdb/releases/chembl_17/chembl_17.sdf.gz

The current web site from data source may or may not present the record version that was reported in Table 4.

For comparing CVSP validation with PubChem we uploaded same DrugBank data set into PubChem. PubChem validation has found 350 errors with name “input record is invalid”, however it appears that they all are raised from standardization phase. Also, it appears that PubChem does not do validation of InChI and Smiles in reference to the provided structure.

### Future work

The CVSP platform has been applied to the analysis of small datasets generally of the order of a few thousand records with the largest collection being the ChEMBL dataset with over million records. As the developers of the ChemSpider database we are conscious of the fact that had we had in place a validation and standardization platform of the nature of CVSP early in the development of the platform that the resulting rigor for automated data review would have resulted in less erroneous data being deposited into the database. While our approach to providing the ability to the community to participate in crowdsourcing a data review process on ChemSpider has been successful, with hundreds of thousands of annotations of the data and significant quantities of user-generated data deposited to the platform, we are conscious that automated review of the data to generate improvements in data quality is still feasible. ChemSpider presently contains over 30 million unique chemical compounds and we will be processing the data through CVSP and analyzing the resulting data to tweak and improve the rules as appropriate.

We believe that numerous rule sets can be developed for various purposes such that CVSP can be a flexible platform serving various purposes. In particular, following conversations with numerous scientists regarding the preparation of files for the purpose of structure-activity analysis (SAR/QSAR) it is possible that the encoding of rules to perform operations such as desalting and neutralization would result in the production of homogeneous files that can be processed by the community. This would be similar in outcome to the adoption and sharing of standard pipelining protocols [24] made available for tools such as Pipeline Pilot [25] or KNIME [26]. For the various projects that the Royal Society of Chemistry is involved in where we are responsible for the hosting of chemical registry systems, such as Open PHACTS [11] or PharmaSea [27], it is possible that different rule sets will be developed for each project.

## Conclusion

The Chemical Validation and Standardization platform (CVSP) has been developed with the intention of providing an environment for the processing of chemical structure files through tested validation and standardization protocols. The intent is to assist the community in the rigorous analysis of their chemical structure files with one specific intention to ensure that data released into the public domain via online databases is pre-validated to the largest extent possible. While the CVSP platform is designed with flexibility inherent to the rules that can be used for processing the data we have produced a recommended rule set based on our own experiences with the analysis of DrugBank and ChEMBL datasets and detected numerous issues within the datasets.

## Dedication

Jean-Claude (JC) Bradley was our often time collaborator, contributor of data to ChemSpider and fellow scientist concerned with data quality online. JC instilled in many of his students a need to question data quality, to adopt standards for data interchange where feasible and, first and foremost, to make data available as Open Data. The Chemical Validation and Standardization Platform described in this work was of interest to JC even in its earliest form. It has been developed further since he originally provided feedback and we believe that at this stage he would be a regular user and would utilize it as one of the tools in his armory for reviewing data. We dedicate this article to his memory.
